# Analysis of Potential Q-Markers for Salt-Processed Alismatis Rhizoma in Diuresis Based on Fingerprinting Technology and Network Analysis

**DOI:** 10.3390/cimb47090783

**Published:** 2025-09-21

**Authors:** Lin Yan, Zemin Ou, Yun Wang, Yan Tong, Jinyu Wang, Dewen Liu

**Affiliations:** 1 Institute of Chinese Materia Medica, China Academy of Chinese Medical Sciences, Beijing 100700, China; yanlin1014@126.com (L.Y.); zeminou@163.com (Z.O.); ywang@icmm.ac.cn (Y.W.); tongyan1012@sohu.com (Y.T.); 2 Experimental Research Center, China Academy of Chinese Medical Sciences, Beijing 100700, China

**Keywords:** Alismatis Rhizoma (*Alisma plantago-aquqtica* L.), diuresis, molecular docking, network analysis, Q-marker, fingerprinting

## Abstract

Introduction: The ability of salt-processed Alismatis Rhizoma (SAR) (*Alisma plantago-aquqtica* L.) to nourish Yin and promote urination is stronger than that of Alismatis Rhizoma (AR). However, there are few studies focused on evaluating the quality of its medicinal materials. Objectives: This study aimed to identify potential quality markers (Q-markers) for SAR, thereby providing a more reliable basis for its quality control and clinical application. Methods: Q-markers were identified through fingerprinting and chemical pattern recognition analysis of 15 batches of SAR. The diuretic effects of these markers were then verified by network analysis and molecular docking. Results: HPLC fingerprints of 15 SAR batches were established, with similarity analysis showing values > 0.85 (0.852–0.990). Chemical pattern recognition identified six critical compounds contributing to SAR quality: alisol F, alisol C 23-acetate, alisol A, alisol A 24-acetate, alisol B 23-acetate, and an alisol O isomer (VIP > 1.0). Network analysis revealed 76 overlapping targets between these compounds and diuretic-related diseases, with core targets including non-receptor tyrosine kinase (SRC), epidermal growth factor receptor (EGFR), mitogen-activated protein kinase 1 (MAPK1), which were identified through protein–protein interaction (PPI) network analysis, with degrees of 27, 24, and 22, respectively. Key pathways involved were the EGFR tyrosine kinase inhibitor resistance pathway, calcium signaling pathway, tumor necrosis factor signaling pathway, etc. Molecular docking confirmed strong binding interactions between the Q-markers and the hub targets, particularly alisol B 23-acetate with MAPK1 (−60.10 kcal·mol^−1^) and alisol A 24-acetate with EGFR (−46.14 kcal·mol^−1^) and SRC (−48.86 kcal·mol^−1^). The diuretic effects of SAR are likely mediated through anti-inflammatory actions and regulation of water–sodium balance via multi-target and multi-pathway mechanisms. Conclusion: This study provides a robust foundation for quality control and clinical application of SAR, though further in vivo validation is warranted.

## 1. Introduction

Alismatis Rhizoma (AR) is the dried tuber of *Alisma plantago-aquqtica* L., a traditional diuretic used in China with a long history of use for treating edema, as seen in formulations like Wulingsan, Baizhusan, and Zexie Decoction. The active ingredients of AR are primarily terpenoids [1]. The 2020 edition of the Pharmacopoeia of the People’s Republic of China (hereinafter referred to as the Chinese Pharmacopoeia) includes two specifications, i.e., AR and salt-processed AR (SAR). Research indicates that both the ethyl acetate and n-butanol fractions of AR exhibit significant diuretic effects, with dual actions of promoting and inhibiting diuresis. The more polar components in AR may have anti-diuretic properties [2]. The total triterpene extract (TTE) and triterpene component compatibility (TCC—a mixture of alisol B 23-acetate, alisol B, alisol A 24-acetate, alisol A, and alisol C 23-acetate) also exhibit significant diuretic effects in saline-loaded rats [3], possibly by enhancing sodium and chloride symporters in the distal tubule, thereby altering the transport rates of sodium, potassium, and chloride ions. This effect is primarily attributed to triterpenes [4].

The Depei Bencao records that raw or wine stir-fried products of AR are used for strengthening the spleen, while salt-processed products are used for nourishing Yin and promoting diuresis. Previous laboratory pharmacological experiments also demonstrated that the diuretic effects of AR were enhanced after salt processing. Both AR and SAR showed therapeutic effects in a rat model of edema due to kidney Yin deficiency, with SAR outperforming AR in restoring related indices. Transcriptomics suggested that the SAR group was more effective than the AR group in regulating inflammation-related targets, potentially explaining efficacy of SAR [5,6]. However, the specific ingredients responsible for this enhancement have not been reported. Most triterpenoid components showed a decreasing trend after AR was salt-processed [7]. The 2020 edition of the Chinese Pharmacopoeia uses alisol C 23-acetate and alisol B 23-acetate as the index components for determining the content of AR and SAR, but these are insufficient to represent the quality control components of AR and SAR. Studies have reported significant diuretic activity of alisol A 24-acetate, alisol B, and other compounds, though whether these ingredients are related to the enhanced efficacy of SAR remains unreported [8].

Chinese medicine quality markers (Q-markers), a new concept proposed by Academician Liu Changxiao in 2016 to improve the quality control of Chinese medicine products, are chemical substances present in Chinese herbal medicines and Chinese medicine products inherently or formed during processing and preparation that are closely related to the functional properties of Chinese medicines and can be used as markers to reflect the safety and efficacy of Chinese medicines, rather than chemical components generated by their metabolic transformation through intra-biological processes [9,10]. The establishment of a Q-marker for traditional Chinese medicine (TCM) is of great significance for quality control and improvement of the quality standards of TCM, which can effectively improve the overall quality evaluation of TCM beverages [11,12,13]. At present, the quality evaluation studies on SAR mainly focus on the trait, alisol B 23-acetate content, and so forth, while the Q-marker has not been explored to date [14].

The fingerprint has the characteristics of integrity and dynamics, which can objectively reflect the ingredients in TCM. Moreover, it can reveal the characteristics, traceability, and measurability of ingredients. Network analysis can comprehensively analyze the relationship between ingredients and the efficacy of prepared slices from the drug–disease–target–pathway network to reflect the effectiveness of ingredients and the rationality of compatibility [15]. Based on this, the present study used fingerprint combined with network analysis research mode to determine the Q-markers of the diuretic enhancement of SAR, aiming to provide a scientific basis for quality control and clinical application of SAR.

## 2. Materials and Methods

### 2.1. Materials

AR products were purchased from Jiangxi Guhan Refined Chinese Medicine Tablets Co., Ltd. (Nanchang, China) and were identified as the dried tubers of *A. plantago-aquqtica* L. in accordance with the Chinese Pharmacopoeia (2020 edition). The identification was conducted by Prof. Qianfeng Gong from the School of Pharmacy at Jiangxi University of Chinese Medicine, Nanchang, China. The details of the prepared slices are presented in Table 1.

### 2.2. Reagents and Instruments

Chemical standards of alisol F (No. CHB160926, 98%) was purchased from Sichuan Chengdu Chroma Biotechnology Co., Ltd. (Chengdu, China). Alisol A 24-acetate (No. Z-061-170417, 98%), alisol B 23-acetate (No. Y-036-171216, 98%), and alisol C 23-acetate (No. Z-062-181015, 98%) were obtained from Chengdu Ruifensi Biotechnology Co., Ltd. (Chengdu, China). Alisol A (No. ST14480120, 98%) was purchased from Shanghai Standards Technical Service Co., Ltd. (Shanghai, China). High-performance liquid chromatography (HPLC)-grade methanol and acetonitrile were purchased from Merck (Darmstadt, Germany). Distilled water was sourced from Watson’s Food & Beverage Co., Ltd. (Guangzhou, China). The instruments used included a BT125D electronic analytical balance (*d* = 0.01 mg, Sartorius Scientific Instruments [Beijing] Co., Ltd., Beijing, China), a KQ-250DB ultrasonic cleaner (Kunshan Ultrasonic instrument Co., Ltd., Kunshan, China), and an e2695 high-performance liquid chromatograph (Waters, Milford, MA, USA).

### 2.3. Methods

#### 2.3.1. Preparation of SAR

Briefly, crude AR slices (2 kg) were soaked in a 2% salt solution (2:100, salt-to-water ratio, *w*/*v*) in a sealed container until fully absorbed. The moistened AR was then stir-fried in a pan at a temperature of 80–120 °C for 10 min, until the water evaporated. After cooling to room temperature, the SAR was obtained and used for further analysis [16,17].

#### 2.3.2. Preparation of Sample Solutions for Fingerprint Analyses

A precisely weighed 1.0 g of SAR was placed in a stoppered Erlenmeyer flask, mixed with 25.0 mL of acetonitrile, sonicated at 100 kHz for 30 min, and then cooled to room temperature. Acetonitrile was added to bring the solution back to its original weight, and the mixture was shaken well. The solution was then filtered through a 0.22 μm membrane to obtain the test solution [18]. A total of 15 batches of SAR were prepared for the test solution.

#### 2.3.3. HPLC Conditions for Fingerprint Analysis

Chromatography was performed using an e2695 liquid chromatograph system (Waters, Milford, USA). All samples of fingerprint analysis were analyzed at a column temperature of 30 °C on a Topsil C_18_ column (4.6 mm × 250 mm, 5 μm). The mobile phase consisted of acetonitrile (A), water (B), and methanol (C). The gradient elution was set as follows: 0–2 min, 35% A, 60% B, and 5% C; 2–5 min, 35–50% A, 60–46% B, and 5–4% C; 5–8 min, 50% A, 46% B, and 4% C; 8–40 min, 50–65% A, 46–32% B, and 4–3% C; 40–75 min, 65% A, 32% B, and 3% C; 75–85 min, 65–90% A, 32–9% B, and 3–1% C; 85–95 min, 90% A, 9% B, and 1% C; 95–96 min, 90–35% A, 9–60% B, and 1–5% C; and 96–105 min, 35% A, 60% B, and 5% C (refer to Supplementary Table S2). The injection volume was 10 µL, with a flow rate of 1.0 mL·min^−1^, and the detection wavelength was set at 210 nm. The similarity analysis was performed using the Similarity Evaluation System for the Chromatographic Fingerprint of TCM software (Version 2012, Chinese Pharmacopoeia Committee, Beijing, China). The fingerprint obtained from the analysis of sample S1 was used as a reference, and the similarity between samples was calculated using multi-point correction (auto-align, time interval 0.3 min) matching.

#### 2.3.4. Chemical Pattern Recognition Analysis

This study analyzed the differences among 15 batches of SAR and screened for the chemical compounds responsible for these differences. The HPLC fingerprints of all 15 batches were imported into SIMCA 13.0 software (Umetrics, Malmö, Sweden) for analysis. Principal component analysis (PCA), hierarchical cluster analysis (HCA), orthogonal partial least-squares discrimination analysis (OPLS-DA), and variable importance in the projection (VIP) analysis were conducted using the peak area of the common peaks as a variable.

#### 2.3.5. Network Analysis

##### Acquisition of Ingredients and Disease Targets

Based on chemical pattern recognition analysis, six compounds were identified as markers of SAR. The Simplified Molecular Input Line Entry System (SMILES) strings of these components were obtained using PubChem (https://pubchem.ncbi.nlm.nih.gov/) [accessed on 20 October 2023] and Swiss ADME (http://www.swissadme.ch/index.php) [accessed on 20 October 2023]. These SMILES strings were then input into Swiss Target Prediction (http://old.swisstargetprediction.ch/) [accessed on 22 October 2023] to predict potential targets for each component. The top 100 targets for each component were selected and summarized. Additionally, “diuresis or edema” was used as a search term in GeneCards (https://www.genecards.org/) [accessed on 24 October 2023] to identify disease-related targets. Disease targets with a relevance score of >2.0 were screened, and intersecting targets between the component targets and disease targets were identified using Venn diagrams.

##### Protein–Protein Interaction (PPI) Network and Enrichment Analysis of Intersection Targets

The intersection targets were entered into STRING (https://cn.string-db.org/) [accessed on 25 October 2023], selecting *Homo sapiens* as the species, with a minimum required interaction score of >0.7. Discrete targets were excluded, and the results were saved in TSV format and visualized using Cytoscape 3.8.0. The 76 intersecting targets were further analyzed using Metascape (https://metascape.org/gp/index.html#/main/step1) [accessed on 25 October 2023], selecting *H. sapiens* as the species. A personalized analysis was conducted, with a filter condition of *p* < 0.01. Gene Ontology (GO) and Kyoto Encyclopedia of Genes and Genomes (KEGG) pathway enrichment analyses were performed, and the results were visualized using the Bioinformatics platform (http://www.bioinformatics.com.cn/) [accessed on 28 October 2023].

#### 2.3.6. Molecular Docking

Molecular docking is a theoretical simulation method used in drug design to explore the interaction between receptors and ligands. It primarily examines intermolecular interactions and predicts binding patterns and affinities [19,20]. The three-dimensional (3D) crystal structures of candidate proteins for binding with the core targets, i.e., non-receptor tyrosine kinase (SRC, PDB ID 3QLG), epidermal growth factor receptor (EGFR, PDB ID 5HG8), and mitogen-activated protein kinase 1 (MAPK1, PDB ID 6SLG), were obtained from the Protein Data Bank (PDB) (http://www.wwpdb.org/) [accessed on 30 October 2023]. The primary criteria for candidate protein selection included: (1) origin from Homo sapiens; (2) higher resolution preferred; (3) pH value close to the normal human physiological range; and (4) structural integrity. Discovery Studio 2020 software (College of Pharmacy, Henan University of Chinese Medicine) was utilized to prepare and optimize the proteins and drug molecules to ensure accuracy and consistency. The process involved obtaining the 3D crystal structures of the target proteins from the PDB database. The proteins were then prepared using the Discovery Studio 2020 software, which involved dehydration, hydrogenation, and the supplementation of amino acid residues. Similarly, the 3D structures of the ligand molecules were transformed and prepared using the prepare ligand program (included hydrogen addition and force field application). The original ligands of the target proteins were used as positive controls to define the active binding pocket. Molecular docking was performed using the CDOCKER program, with a pose cluster radius set to 0.5 and other parameters left at default for virtual calculations. The root mean square deviation (RMSD) of the ligand was calculated to ensure that the calculated results accurately simulate the binding between the original ligands and proteins. An RMSD value of less than 2 indicates that the results are reliable. Additionally, the suitability of the docking procedure and parameters for the receptor proteins was verified. Molecular docking results were evaluated based on -CDOCKER INTERACTION ENERGY scores, interaction sites, and interaction types. Meanwhile, using the LibDock program based on the aforementioned active pocket parameters, and the LibDockScores were used for result evaluation.

## 3. Results

### 3.1. Validation of the Fingerprint Method

The alisol B 23-acetate, with a prominent peak area in the chromatogram, was set as the reference peak (peak 10). Relative retention times (RRTs) and relative peak areas (RPAs) were calculated, and the fingerprint method was verified using these indicators.

Precision, a prerequisite for ensuring measurement accuracy, was assessed by repeatedly injecting the same sample six times. A SAR sample was prepared to obtain the sample solution, which was then analyzed continuously six times according to the chromatographic conditions outlined in Section 2.3.3. The relative standard deviations (RSDs) of RRTs for the common peaks ranged from 0.01% to 0.09%, and the RSDs of RPAs ranged from 0.07% to 2.58%. Repeatability was evaluated by having the same operator prepare and test six samples in the same laboratory and under the same conditions. For the same SAR sample, six parallel samples were prepared according to Section 2.3.2 and analyzed. The RSDs of RRTs for common peaks ranged from 0.02% to 0.19%, and the RSDs of RPAs ranged from 0.85% to 2.46%. Stability was assessed by evaluating the same sample at multiple time points. The SAR sample solution was tested at 0, 2, 4, 8, 12, and 24 h after preparation. The RSDs of RRTs for common peaks ranged from 0.03% to 0.22%, and the RSDs of RPAs ranged from 1.09% to 2.65%, indicating that the sample remained stable for 24 h.

The results demonstrate that the sample remained stable throughout the experiment and that the fingerprint analysis method for SARs was both reliable and reproducible.

### 3.2. Similarity Analysis

**Figure 1:**
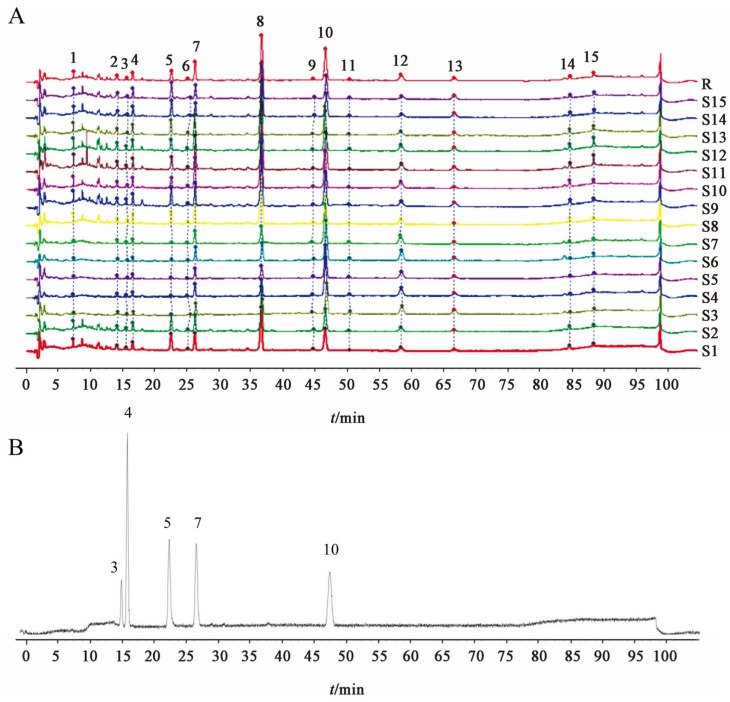
HPLC fingerprints of 15 batches of SAR samples (**A**) and mixed reference solution (**B**), peak 3: alisol F, peak 4: alisol C 23-acetate, peak 5: alisol A, peak 7: alisol A 24-acetate, peak 10: alisol B 23-acetate.

The thumbnail similarity results showed that the similarity between each sample and the control fingerprint was >0.85 (Table 2, Figure 1A,B). Most samples exhibited a similarity > 0.9, though some samples (S3–S7) had a similarity < 0.9. It was observed that SARs from different manufacturers and origins showed similar chromatographic profiles, with 15 major shared peaks identified.

### 3.3. Chemical Pattern Recognition Analysis

**Figure 2:**
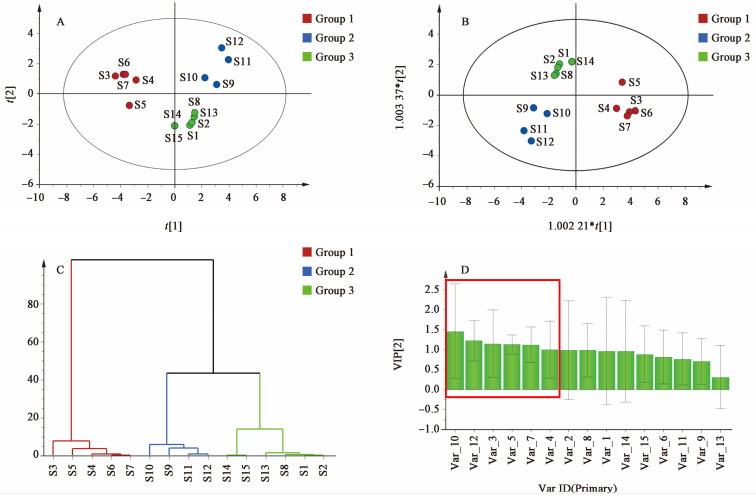
Analysis of PCA scores (**A**), OPLS-DA (**B**), HCA (**C**), and VIP values (**D**) of SAR. The red box in subfigure (**D**) indicates the section where the VIP values exceed 1.0.

As shown in Figure 2, the peak area of the common peak in the samples was used as the evaluation index, and the grouping of the samples was consistent with the similarity analysis. The results of PCA (Figure 2A), HCA (Figure 2B), and OPLS-DA (Figure 2C) were aligned, indicating that the 15 batches of SAR could be divided into three groups: Group 1 (S3–S7), Group 2 (S10–S12), and Group 3 (S1, S2, S8, and S13–S15). Group 1 exhibited a similarity of <0.9, while Groups 2 and 3 had similarities of >0.96. Further analysis suggested that the differences might be related to the origin of the medicinal materials and the production enterprises. Samples S3–S7, all purchased from Jiangxi Guhan Refined Chinese Medicine Tablets Co., Ltd., had a low overall similarity but showed a similarity of >0.99 among themselves. These samples were from the same origin (Guangchang, Jiangxi), indicating that the production enterprises had a lower impact than the place of origin on the quality of the medicinal materials. The OPLS-DA analysis demonstrated a high fitting degree and good predictive ability (R2X: 0.755, R2Y: 0.893, *Q*^2^: 0.802). Based on VIP > 1.0 (Figure 2D), peaks 10, 12, 3, 5, 7, and 4 were identified as having a significant impact on the quality evaluation of SAR and could potentially serve as Q-markers. Preliminary identification of compounds corresponding to peaks 3, 4, 5, 7, 10, and 12 was conducted using laboratory standards. Peaks 3, 4, 5, 7, and 10 were identified as alisol F, alisol C 23-acetate, alisol A, alisol A 24-acetate, and alisol B 23-acetate, respectively. Peak 12 was identified as an alisol O isomer, which was structurally analyzed using retention time, precise molecular weight, and MS/MS data obtained by UPLC–QTOF–MS/MS (refer to Supplementary Table S1 and Supplementary Figures S1–S7 for details ).

### 3.4. Network Analysis

#### 3.4.1. Ingredients and Disease Targets

**Figure 3:**
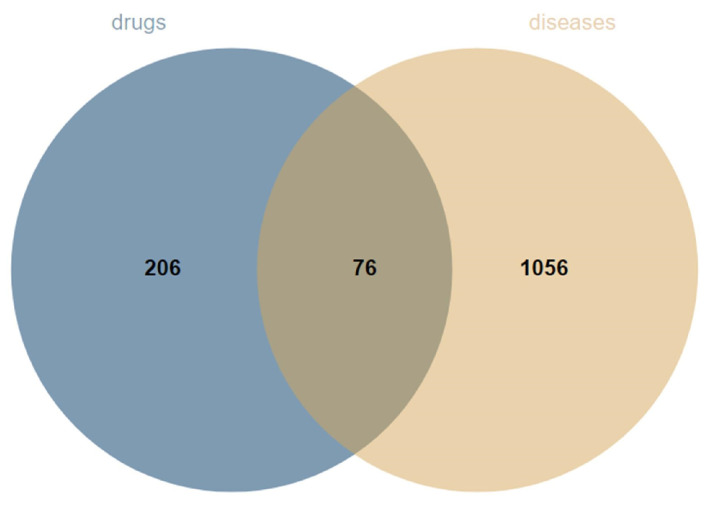
Venn diagram of overlapping targets of SAR and edema.

From the database search, the top 100 targets for each component were summarized, and redundant targets were removed, resulting in a total of 282 targets. Subsequently, 1132 disease targets with a high correlation (relevance score > 2.0) were screened, leading to the identification of 76 intersecting targets that served as both component and disease targets (Figure 3).

#### 3.4.2. PPI Network

**Figure 4:**
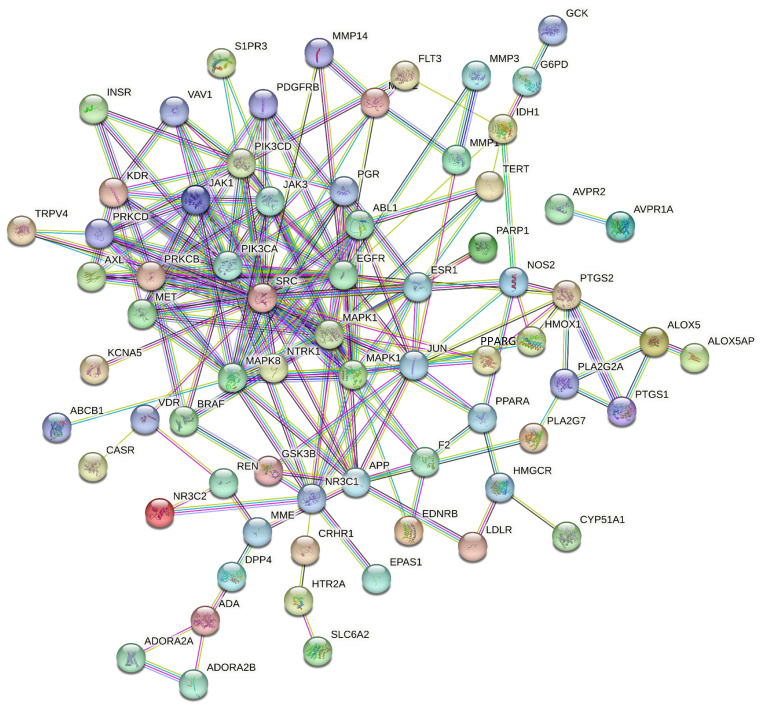
PPI network of 68 genes.

**Figure 5:**
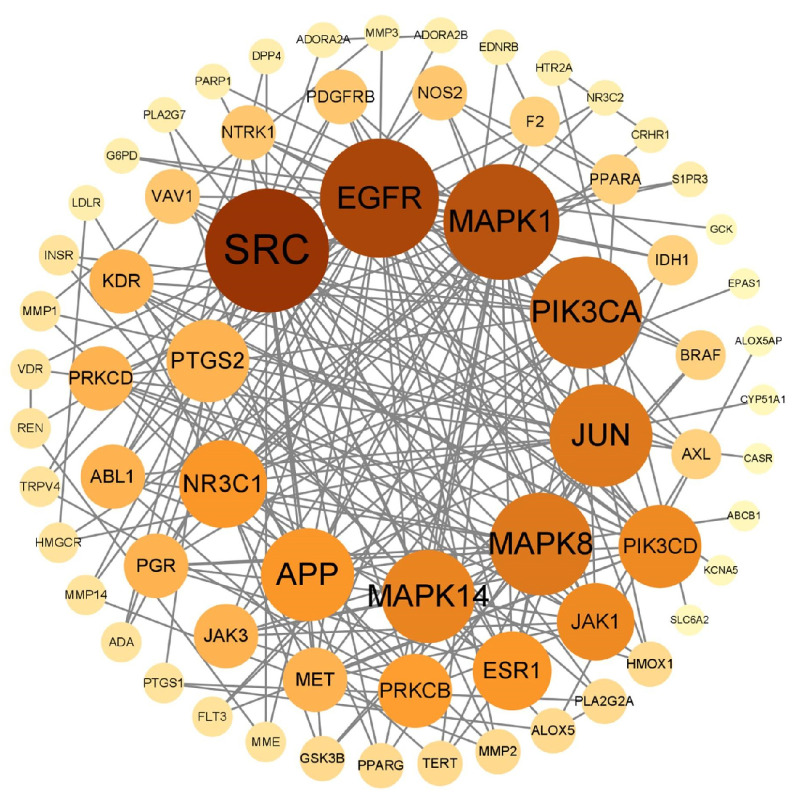
Visual screening of core targets by Cytoscape 3.8.0.

PPI network comprised 68 nodes and 203 edges. In the network, nodes with higher degree values and darker colors indicate greater importance. Topological analysis using the CytoNCA plug-in and “Network Analyzer” revealed average values for degree centrality, betweenness centrality, and closeness centrality of 5.97, 136.32, and 0.35, respectively. Ten targets exceeded the mean values for degree centrality, betweenness centrality, and closeness centrality. These core targets, including SRC, EGFR, and MAPK1, were highlighted (Figure 4 and Figure 5, Table 3).

#### 3.4.3. Enrichment Analysis

**Figure 6:**
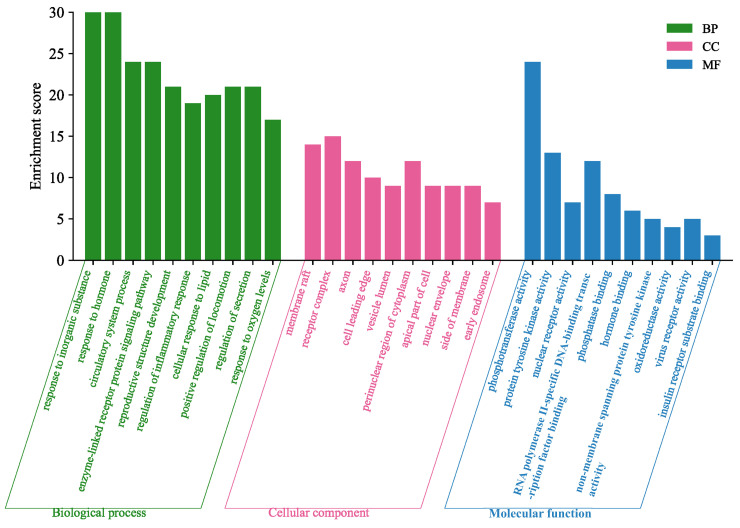
GO function enrichment analysis (top 10).

**Figure 7:**
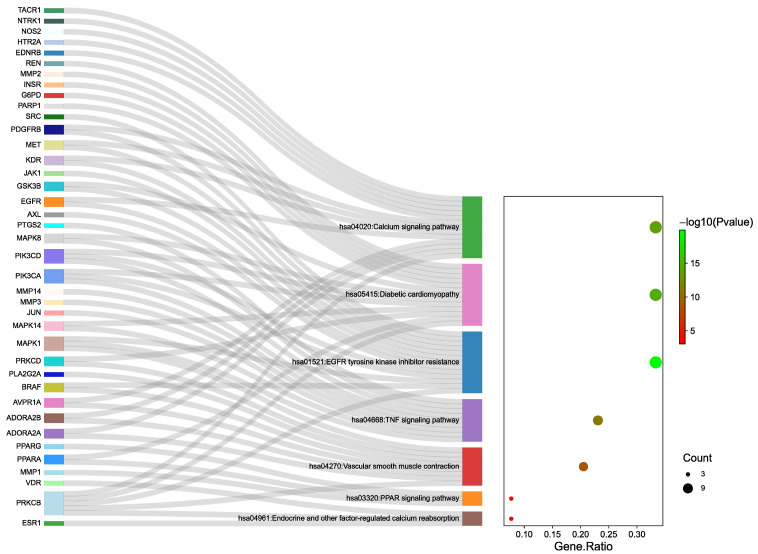
KEGG pathway enrichment analysis.

The GO analysis identified 999 biological processes (BP), 97 molecular functions (MF), and 65 cellular components (CC), with the top 10 entries visually displayed (Figure 6). The KEGG pathway enrichment analysis revealed a total of 150 pathways, from which the 17 most significant pathways were analyzed. Among these, seven pathways that were most directly related to diuresis were selected for visualization (Figure 7, Table 4). The BP analysis primarily involved the regulation of inflammatory responses, hormonal reactions, and circulatory system processes. The CC analysis was related to membrane rafts, receptor complexes, and axons. The MF analysis suggested that the diuretic effects of SAR might be associated with phosphotransferase activity, protein tyrosine kinase activity, and nuclear receptor activity. The above results indicate that the diuretic activity of the six main components could be mediated through multiple pathways and targets.

#### 3.4.4. Component–Target–Pathway Network

**Figure 8:**
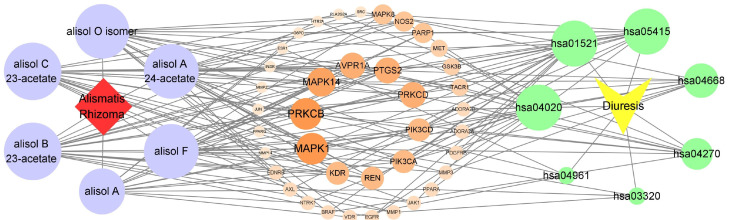
“Component–target–pathway” network. Purple circles denote quality markers, orange circles represent targets, and teal circles indicate pathways. The size and color intensity of each circle reflect its significance and relevance, respectively. Larger sizes and darker shades represent greater importance and stronger associations.

The network diagram illustrating the “component–target–pathway” relationship was plotted by linking the 76 targets associated with the diuretic effects of the six essential components of SAR with the seven key diuretic pathways (Figure 8). As shown in the figure, the key pathways involved in the diuretic effects include the EGFR tyrosine kinase inhibitor resistance pathway, calcium signaling pathway, and tumor necrosis factor (TNF) signaling pathway. Studies indicate that these pathways are involved in inflammation, aquaporin regulation, and other processes, playing a crucial role in the anti-inflammatory immune response. This suggests that the diuretic effects of SAR may be mediated by reducing renal inflammation and improving the balance of water and sodium.

### 3.5. Molecular Docking

Molecular docking was conducted to investigate the binding of the six Q-markers to three target proteins [SRC (PDB ID: 3QLG), EGFR (PDB ID: 5HG8), and MAPK1 (PDB ID: 6SLG)]. The CDOCKER-INTERACTION-ENERGY module in Discovery Studio software was used to verify the effectiveness of these compounds and elucidate the mechanism of SAR in treating edema. The affinity and binding modes of the components with the target proteins were assessed based on the docking results. The RMSD values for all dockings were less than 2.0, indicating reliable docking predictions (Table 5, Figure S8). Higher -CDOCKER-INTERACTION-ENERGY and LibDockScore values corresponded to stronger binding activity between the compounds and proteins. The interaction energy scores are detailed in Table 6. Notably, alisol B 23-acetate exhibited the highest affinity with MAPK1, as evidenced by its highest -CDOCKER-INTERACTION-ENERGY value of 60.104 4.

**Figure 9:**
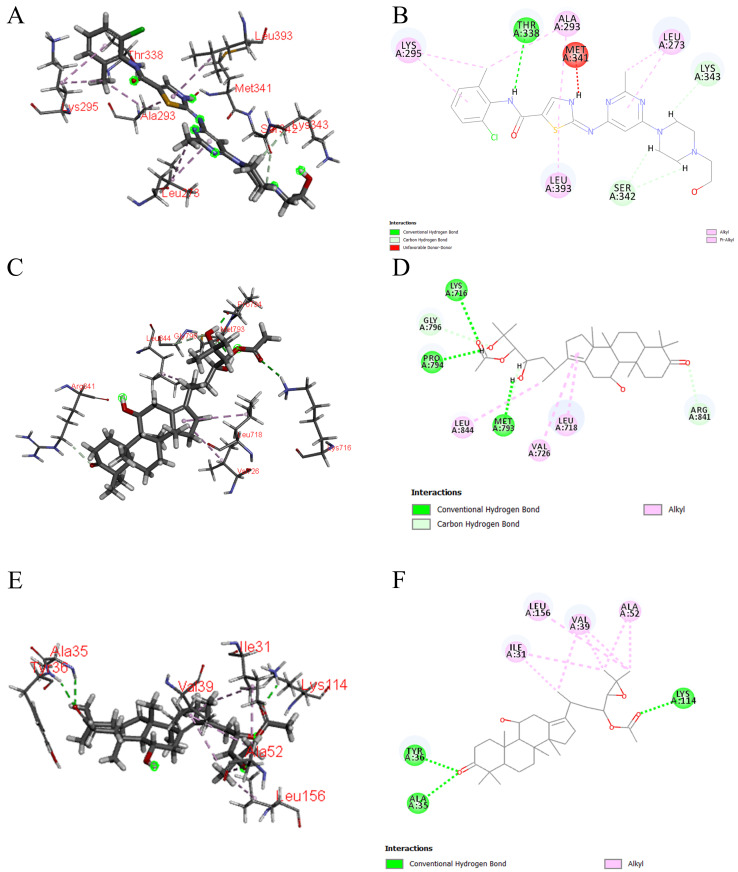
Molecular docking models of alisol A 24-acetate with SRC and EGFR, and alisol B 23-acetate with MAPK1 in 3D and 2D diagrams. 3D and 2D diagrams of alisol A 24-acetate with SRC (**A**,**B**); alisol A 24-acetate with EGFR (**C**,**D**); alisol B 23-acetate with MAPK1 (**E**,**F**).

Figure 9A–F illustrate the molecular docking models of the compounds with the target proteins, presented in both 3D and 2D diagrams. The results demonstrated that all ligand compounds effectively bound to the active pockets of the proteins. Specifically, alisol B 23-acetate interacted with amino acid residues LYS114, TYR36, and ALA35 through hydrogen bonds formed by its ester and carbonyl groups (Figure 9E,F). Meanwhile, alisol A 24-acetate formed hydrogen bonds with residues MET793, LYS716, and PRO794 via its hydroxyl group (Figure 9C,D). Additionally, non-covalent bonds between the ester and carbonyl groups and amino acid residues ARG841 and GLY796 were observed (Figure 9C,D). These interactions reduced binding energy and enhanced affinity. The candidate monomers were tightly bound to the active sites of the proteins, forming stable complexes with strong binding activity (Table 7). These findings suggest that the six Q-markers may possess significant biological activity, providing a basis for quality control of SAR.

## 4. Discussion

SAR, serving as the most commonly used processed variety, underscores the importance of establishing quality standards for its effective development and use. Q-markers offer valuable references for quality control of SAR. By utilizing fingerprinting technology, this study identified common peaks in SAR from different origins, screened compounds with significant contributions through chemical pattern recognition, and analyzed the diuretic mechanisms of these compounds via network analysis and molecular docking. This comprehensive evaluation of Q-markers for SAR aims to establish quality standards, provide data support for clinical applications, and ultimately better serve patients with renal edema.

The fingerprint analysis of 15 batches of SAR identified 15 compounds with high content across different manufacturers and origins. This suggests that the content of these ingredients significantly affects the efficacy of SAR. VIP analysis highlighted six key compounds (peaks 3, 4, 5, 7, 10, and 12) with VIP values of >1.0 as potential Q-markers for SAR. These compounds include alisol F, alisol C 23-acetate, alisol A, alisol A 24-acetate, alisol B 23-acetate, and an alisol O isomer. Pharmacological research supports the significant activity of these compounds. Alisol F, alisol A, and alisol A 24-acetate are known for their anti-inflammatory effects by inhibiting inflammatory cytokines [21,22], making them crucial in treating nephrotic edema. Alisol C 23-acetate and alisol B 23-acetate, which are used as index components in the content determination of AR, also demonstrate anti-inflammatory, diuretic, and lipid-lowering activities [3,23]. Specifically, alisol B 23-acetate has been shown to activate renal farnesoid X receptor (FXR), providing renoprotection against ischemia–reperfusion injury and alleviating renal fibrosis through mechanisms such as gut microbiome modulation and blood pressure regulation [24,25]. This indicates its significant potential in treating renal diseases. Although alisol O exhibits nephrotoxicity [24], the activity of its isomer remains unexplored, warranting further investigation to determine if it also possesses nephrotoxic effects.

Edema is commonly associated with various pathological conditions such as nephritis and nephrotic syndrome, leading to sodium retention and microcirculation disorders. Inflammation in the body is a significant predisposing factor for edema [25], as it can lead to renal edema through mechanisms such as renal neutrophil influx and capillary leakage [26]. Therefore, reducing inflammation and enhancing the body’s immune response are crucial for treating edema and may be key to the diuretic effects of certain drugs. Research has demonstrated that alisol F and 25-anhydroalisol F inhibit the production of nitric oxide (NO), interleukin-6 (IL-6), tumor necrosis factor-alpha (TNF-α), and interleukin-1β (IL-1β) by interfering with the MAPK, STAT3, and NF-κB signaling pathways in cells [21]. MAPK, a crucial upstream signaling molecule for NF-κB, is implicated in the core targets of SAR for edema treatment. As an essential pathway of intracellular signal transduction, MAPK is involved in regulating cell growth, oxidative stress, inflammatory responses, and other cellular processes. Chae, Song [27] found that inhibiting MAPK signaling phosphorylation and activating degranulation modulated the expression of inflammatory mediators. Pathway enrichment analysis further highlighted MAPK as a significant target within the TNF signaling pathway, with MAPK1, MAPK14, and other related proteins playing key roles in this pathway.

In addition, this study suggests that SAR may also exert anti-inflammatory and immune-modulating effects through targets such as SRC and EGFR, along with their associated pathways. SRC is known to regulate various signaling pathways related to biological activities, including gene transcription, immune responses, and cell adhesion. Inhibiting SRC activity can reduce the release of pro-inflammatory cytokines, thereby ameliorating inflammatory responses [28]. EGFR, a receptor for epidermal growth factor and transforming growth factor α, interacts with active ingredients in SAR to block the binding of pro-inflammatory factors, thereby exerting anti-inflammatory effects and enhancing the body’s immune function [29]. Moreover, KEGG enrichment analysis indicated that the EGFR tyrosine kinase inhibitor resistance pathway might be involved in the diuretic effects of SAR. EGFR tyrosine kinase inhibitors mediate multiple signal transduction pathways, transmitting extracellular signals into the cell and playing a crucial regulatory role in the proliferation, differentiation, and apoptosis of normal and tumor cells. This process can enhance the immune response and significantly improve the body’s disease resistance [30]. Research has shown that compounds like alisol F, alisol A, and alisol B 23-acetate can regulate MAPK activation [31,32], thereby inhibiting MAPK expression and activity [21,33,34]. The SRC/phosphatidylinositol 3-kinase (PI3K)/protein kinase B (Akt) signaling pathway is also critical in the pathogenesis of renal fibrosis [35]. Both alisol A 24-acetate and alisol B 23-acetate have been found to inhibit the PI3K/Akt/mammalian target of rapamycin (mTOR) signaling pathway [36]. Since SRC regulates PI3K/Akt signaling, with inhibitors like PP1 and LY294002 inhibiting Akt phosphorylation, it is hypothesized that the components in SAR may act on these inflammatory targets. By exerting anti-inflammatory effects, these components may improve renal inflammation and consequently enhance the balance of water and sodium in the kidneys, thereby achieving diuretic effects.

The GO enrichment analysis indicated that SAR exerted its diuretic effects by modulating inflammatory responses, hormone signaling, and circulatory processes. There appears to be a synergistic interaction among various signaling pathways, as shown by the KEGG pathway enrichment data, which highlighted the involvement of pathways such as EGFR tyrosine kinase inhibitor resistance, peroxisome proliferator-activated receptor (PPAR) signaling, and TNF signaling. Additionally, KEGG analysis revealed that the diuretic effects of SAR may be mediated through pathways like the calcium signaling pathway, endocrine and other factor-regulated calcium reabsorption, and vascular smooth muscle contraction. Research on the diuretic effects of AR in saline-loaded rats demonstrated significant diuretic effects by enhancing the excretion of Na^+^, K^+^, and Cl^−^ ions [37], suggesting that the diuretic effects of AR are closely related to water and sodium ion channels. Studies have shown that calcium signaling pathways may be involved in the activation of diuretic hormones [38]. In vivo experiments with olive leaf extract treatment revealed increased expression of calcium-sensitive receptor mRNA, decreased expression of aquaporin (AQP)-2 mRNA, and increased expression of AQP-2-targeted MicroRNA (miRNA)-137, suggesting that olive leaf extract antagonizes the effects of vasopressin by stimulating calcium-sensitive receptors [39]. In earlier studies by this research group, it was also found that AR and SAR reduced the transcription levels of renal AQP-1 and AQP-2 mRNA, alleviating edema symptoms in rats with kidney Yin deficiency [40]. This effect may be achieved by stimulating calcium-sensitive receptors. The endocrine and other factor-regulated calcium reabsorption pathway plays a key role in regulating intracellular calcium ion concentration, which serves as a secondary messenger in the antidiuretic hormone response [41], directly affecting renal water reabsorption. It is speculated that SAR may act on the calcium signaling pathway, thereby affecting the expression of water and sodium transport proteins and regulating the balance of water metabolism. However, the key targets within these pathways still require experimental validation, and more detailed mechanisms need further investigation.

Moreover, molecular docking results indicated that the six Q-markers effectively bind to core targets, suggesting that the active components of SAR might exert their diuretic effects by participating in EGFR tyrosine kinase inhibitor resistance, regulated by EGFR. Consequently, the Q-markers identified through fingerprinting combined with chemical pattern recognition analysis were validated to reflect their biological activity, as confirmed by network analysis and molecular docking studies. This study provides a solid foundation for the quality control of SAR.

Despite the comprehensive identification of potential Q-markers and the proposed multi-target mechanisms underlying the diuretic effects of SAR, this study has several limitations. Primarily, the network pharmacology and pathway enrichment analyses are predictive in nature and lack experimental validation, particularly through in vivo models, to confirm the actual involvement of targets such as MAPK, SRC, EGFR, and calcium signaling pathways in the diuretic action of SAR. Moreover, molecular docking results, while indicative of binding potential, may not fully reflect the actual binding affinity or functional impact of the compounds on the target proteins due to limitations in simulating physiological conditions. Additionally, the current approach does not account for potential synergistic effects among the multiple active compounds in SAR, which could be critical for its overall efficacy. Future studies should prioritize in vivo validation using animal models of renal edema to verify the predicted targets and pathways, incorporate binding affinity assays (e.g., surface plasmon resonance) to confirm compound–target interactions, and explore combinatory effects of the Q-markers to assess synergism. Furthermore, detailed mechanistic studies, including gene knockout or pharmacological inhibition of key targets, would help elucidate the precise contributions of each pathway to the diuretic activity of SAR.

## 5. Conclusions

This study successfully identified six potential Q-markers in SAR through fingerprinting and chemical pattern recognition, and proposed a multi-target mechanism for their diuretic effects involving anti-inflammatory and ion transport regulation, primarily via MAPK, EGFR, and calcium signaling pathways, as supported by network pharmacology and molecular docking. However, these predictions require further experimental validation using in vivo models and binding affinity assays to confirm target engagement and functional effects. Future research should also explore potential synergies among the Q-markers and elucidate detailed mechanisms through pharmacological inhibition or genetic manipulation of key targets, ultimately contributing to more reliable quality control and clinical application of SAR.

## Figures and Tables

**Table 1:** Details of prepared slices.

Manufacturer	Number	Origin	Lot Number
Jiangxi Guhan Refined Chinese Medicine Tablets Co., Ltd. (Nanchang, China)	S1	Jiangxi	20180401
	S2	Jiangxi	20180501
	S3	Jiangxi	20180706
	S4	Jiangxi	20180707
	S5	Jiangxi	20180708
	S6	Jiangxi	20180709
	S7	Jiangxi	20180710
Beijing Sanhe Pharmaceutical Co., Ltd. (Beijing, China)	S8	Fujian	180908
	S9	Fujian	81221001
Jiangxi Jiangzhong Traditional Chinese Medicine Co., Ltd. (Nanchang, China)	S10	Sichuan	180515
	S11	Sichuan	181031
	S12	Sichuan	180902
Jiangxi Zhangshu Tianqitang Traditional Chinese Medicine Co., Ltd. (Nanchang, China)	S13	Jiangxi	1811010
	S14	Jiangxi	1811011
Zhangshu Qingren Traditional Chinese Medicine Co., Ltd. (Nanchang, China)	S15	Jiangxi	181001

**Table 2:** The similarity of 15 batches of SAR samples.

Sample	S1	S2	S3	S4	S5	S6	S7	S8	S9	S10	S11	S12	S13	S14	S15	Control
S1	1.000	0.999	0.782	0.783	0.765	0.779	0.760	0.986	0.996	0.981	0.986	0.983	0.997	0.989	0.989	0.984
S2	0.999	1.000	0.774	0.773	0.755	0.770	0.750	0.990	0.997	0.984	0.988	0.986	0.998	0.988	0.988	0.983
S3	0.782	0.774	1.000	0.991	0.986	0.992	0.988	0.727	0.790	0.742	0.751	0.759	0.800	0.821	0.821	0.870
S4	0.783	0.773	0.991	1.000	0.998	0.998	0.998	0.728	0.788	0.745	0.750	0.757	0.799	0.813	0.813	0.871
S5	0.765	0.755	0.986	0.998	1.000	0.997	0.999	0.708	0.768	0.725	0.729	0.735	0.781	0.794	0.794	0.855
S6	0.779	0.770	0.992	0.998	0.997	1.000	0.997	0.725	0.782	0.743	0.747	0.754	0.795	0.807	0.807	0.868
S7	0.760	0.750	0.988	0.998	0.999	0.997	1.000	0.703	0.764	0.720	0.724	0.731	0.776	0.790	0.790	0.852
S8	0.986	0.990	0.727	0.728	0.708	0.725	0.703	1.000	0.989	0.997	0.995	0.994	0.988	0.964	0.964	0.969
S9	0.996	0.997	0.790	0.788	0.768	0.782	0.764	0.989	1.000	0.985	0.990	0.989	0.999	0.990	0.990	0.987
S10	0.981	0.984	0.742	0.745	0.725	0.743	0.720	0.997	0.985	1.000	0.997	0.998	0.985	0.954	0.954	0.972
S11	0.986	0.988	0.751	0.750	0.729	0.747	0.724	0.995	0.990	0.997	1.000	1.000	0.990	0.964	0.964	0.976
S12	0.983	0.986	0.759	0.757	0.735	0.754	0.731	0.994	0.989	0.998	1.000	1.000	0.989	0.962	0.962	0.977
S13	0.997	0.998	0.800	0.799	0.781	0.795	0.776	0.988	0.999	0.985	0.990	0.989	1.000	0.991	0.991	0.990
S14	0.989	0.988	0.821	0.813	0.794	0.807	0.790	0.964	0.990	0.954	0.964	0.962	0.991	1.000	1.000	0.982
S15	0.989	0.988	0.821	0.813	0.794	0.807	0.790	0.964	0.990	0.954	0.964	0.962	0.991	1.000	1.000	0.982
Control	0.984	0.983	0.870	0.871	0.855	0.868	0.852	0.969	0.987	0.972	0.976	0.977	0.990	0.982	0.982	1.000

**Table 3:** Core target results.

No.	Targets	Degree Centrality	Betweenness Centrality	Closeness Centrality
1	SRC	27	950.4918	0.496296
2	EGFR	24	978.5427	0.519380
3	MAPK1	22	564.3282	0.492647
4	PIK3CA	18	248.4000	0.432258
5	JUN	16	477.7668	0.482014
6	MAPK8	16	243.1015	0.465278
7	MAPK14	14	173.2342	0.435065
8	NR3C1	11	656.6416	0.429487
9	APP	11	887.3545	0.455782
10	PTGS2	8	462.2155	0.385057

**Table 4:** KEGG pathway enrichment results.

Pathway	*p* Value	Gene ID
hsa04020: Calcium signaling pathway	3.54813 × 10^−14^	ADORA2A, ADORA2B, AVPR1A, EDNRB, EGFR, HTR2A, KDR, MET, NOS2, NTRK1, PDGFRB, PRKCB, TACR1
hsa05415: Diabetic cardiomyopathy	4.0738 × 10^−15^	PARP1, MAPK14, G6PD, GSK3B, INSR, MMP2, PIK3CA, PIK3CD, PPARA, PRKCB, PRKCD, MAPK8, REN
hsa01521: EGFR tyrosine kinase inhibitor resistance	1.28825 × 10^−20^	AXL, BRAF, EGFR, GSK3B, JAK1, KDR, MET, PDGFRB, PIK3CA, PIK3CD, PRKCB, MAPK1, SRC
hsa04668: TNF signaling pathway	1.09648 × 10^−11^	MAPK14, JUN, MMP3, MMP14, PIK3CA, PIK3CD, MAPK1, MAPK8, PTGS2
hsa04270: Vascular smooth muscle contraction	1.77828 × 10^−9^	ADORA2A, ADORA2B, AVPR1A, BRAF, PLA2G2A, PRKCB, PRKCD, MAPK1
hsa03320: PPAR signaling pathway	0.000912011	MMP1, PPARA, PPARG
hsa04961: Endocrine and other factor-regulated calcium reabsorption	0.000331131	ESR1, PRKCB, VDR

**Table 5:** Molecular docking parameters and details.

Proteins	Ligand	Pocket Radius/Å	Coordinates	RMSD	-Cdocker Interaction Energy (kcal·mol^−1^)
3QLG	1N1601	11.86	11.199596, −37.800519, −5.221942	0.5778	56.857
5HG8	6349001	7.96	13.830411, −4.244731, −31.864270	1.2365	46.1437
6SLG	LHZ401	9.77	−3.029314, 5.883335, 12.620670	0.2716	67.3954

**Table 6:** Molecular docking results.

Target	PDB ID	Small Molecule	-Cdocker Interaction Energy (kcal·mol^−1^)	LibDockScore
SRC	3QLG	alisol F	36.4889	106.164
SRC	3QLG	alisol C 23-acetate	42.384	114.835
SRC	3QLG	alisol A	42.3821	112.757
SRC	3QLG	alisol A 24-acetate	48.8646	112.202
SRC	3QLG	alisol B 23-acetate	38.4604	115.884
SRC	3QLG	alisol O isomer	35.1787	107.847
EGFR	5HG8	alisol F	42.0202	99.9377
EGFR	5HG8	alisol C 23-acetate	44.1925	110.194
EGFR	5HG8	alisol A	41.4245	109.214
EGFR	5HG8	alisol A 24-acetate	46.1437	109.635
EGFR	5HG8	alisol B 23-acetate	44.4599	114.636
EGFR	5HG8	alisol O isomer	41.2246	104.867
MAPK1	6SLG	alisol F	52.4389	127.727
MAPK1	6SLG	alisol C 23-acetate	57.6403	135.748
MAPK1	6SLG	alisol A	51.3225	118.283
MAPK1	6SLG	alisol A 24-acetate	58.624	130.905
MAPK1	6SLG	alisol B 23-acetate	60.1044	125.271
MAPK1	6SLG	alisol O isomer	52.0498	113.233

Note: SRC denotes a non-receptor tyrosine kinase, EGFR denotes a epidermal growth factor receptor, and MAPK1 denotes a mitogen-activated protein kinase 1.

**Table 7:** Interaction sites and types of covalent bonds between representative Q-markers and key Proteins.

Target (Small Molecule)	Conventional Hydrogen Bond	Carbon Hydrogen Bond	Alkyl/Pi-Alkyl	Unfavorable Donor–Donor
SRC (alisol A 24-acetate)	Thr338	Lys343, Ser342	Leu393, Leu273, Ala293	Met341
EGFR (alisol A 24-acetate)	Met793, Pro794, Lys716	Arg841, Gly796	Leu718, Val726, Leu844	-
MAPK1 (alisol B 23-acetate)	Tyr36, Ala35, Lys114	-	Leu156, Ile31, Val39, Ala52	-

Note: “-“ Indicates no data.

## Data Availability

The datasets generated and/or analyzed during the current study are available from the corresponding author on reasonable request.

## Supplementary Materials

The following supporting information can be downloaded at: https://www.mdpi.com/article/10.3390/cimb47090783/s1.

## Abbreviations

3D, three-dimensional; AKI, acute kidney injury; Akt, protein kinase B; AQP, aquaporin; AR, Alismatis Rhizoma; BP, biological processes; CC, cellular components; EGFR, epidermal growth factor receptor; FXR, farnesoid X receptor; GO, gene ontology; HCA, hierarchical cluster analysis; IL-1β, Interleukin-1β; IL-6, Interleukin-6; KEGG, Kyoto encyclopedia of genes and genomes; MAPK1, mitogen-activated protein kinase 1; MF, molecular function; MiRNA, MicroRNA; NF-κB, nuclear factor kappa-B; OPLS-DA, orthogonal partial least-squares discrimination analysis; PCA, principal component analysis; PDB, Protein Structure Database; PI3K, phosphatidylinositol 3-kinase; PPAR, peroxisome proliferator-activated receptor; PP1, phosphatase inhibitors; PPI, Protein–protein Interaction Networks; Q-markers, quality markers; RMSD, root mean square deviation; RPAs’, relative peak areas; RRTs’, relative retention times; RSDs, relative standard deviations; SAR, salt-processed Alismatis Rhizoma; SRC, non-receptor tyrosine kinase; STAT3, signal transducer and activator of transcription; TCM, traditional Chinese medicine; TCC, triterpene component compatibility; TNF, tumor necrosis factor; TTE, total triterpene extract; VIP, Variable importance in the projection.
