# Supplementary material for: Analysis of Potential Q-Markers for Salt-Processed Alismatis Rhizoma in Diuresis Based on Fingerprinting Technology and Network Analysis
Source: Curr Issues Mol Biol. 2025 Sep 21;47(9):783. doi: 10.3390/cimb47090783 (PMC12468721; doi:10.3390/cimb47090783)
Supplement: Supplementary file 1 [file cimb-47-00783-s001.zip › cimb-3866738-supplementary.pdf]

UPLC–QTOF–MS/MS analysis

A batch of sample S1 was selected and separated using ACQUITY UPLC BEH C18 (2.1 × 100 mm2, 1.7 μm) column. The parameters were as follows: flow rate 0.3 mL/min; injection volume 2 μL; column temperature 35°C; mobile phase: acetonitrile (A)–0.1% formic acid–water (B); gradient conditions: 0–3 min, 10%–30% A; 3–18 min, 30%–40% A; 18–40 min, 40%–85% A; 40–40.1 min, 85%–10% A; and 40.1–43 min, 10% A. Mass spectrometric analyses were performed on a Waters Xevo G2-S Q-TOF system with an electrospray ionization source, and the positive ion mode was used for data acquisition. The following MS conditions were set: cone gas flow, 50 L/h; desolvation gas flow, 600 L/h; desolvation temperature, 400°C; source temperature, 120°C; cone voltage, 40 V; and capillary voltage, 2.2 kV. The collision energy was set at 15–45 eV. The mass ranges were set at m/z 100–1200 for a full scan with a scan duration of 1 s. The raw spectra data were acquired and managed using MassLynx software (Waters Corporation, MA, USA).

The chemical composition of SAR was analyzed using UPLC–QTOF–MS/MS, and the peaks 3, 4, 5, 7, 10, and 12 were structurally analyzed using retention time, precise molecular weight, and MS/MS data. The base peak ion (BPI) chromatograms of the SAR extracts in the positive mode are shown in Figure 1.

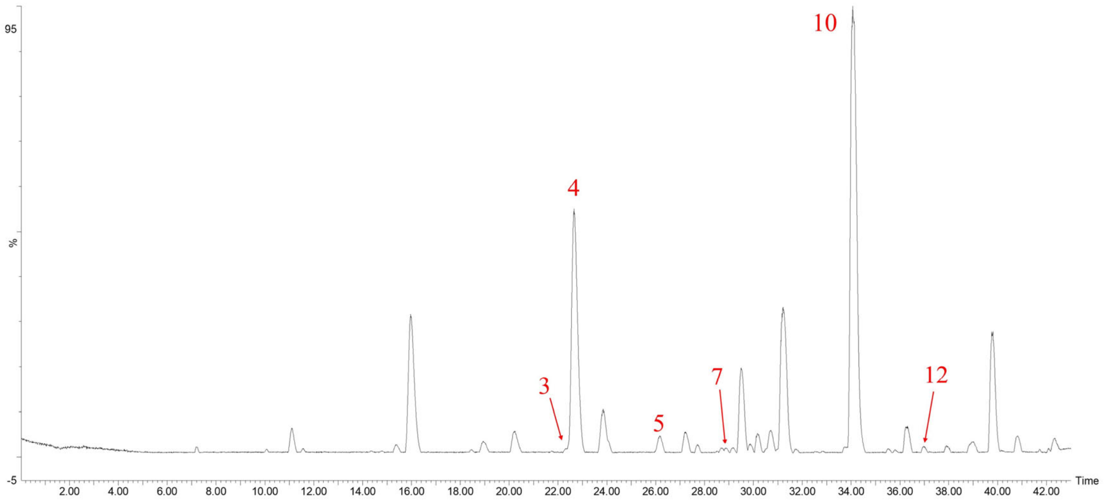

Figure s1. BPI chromatograms of SAR in the positive mode. Peak 3: alisol F; peak 4: alisol C 23-acetate; peak 5: alisol A; peak 7: alisol A 24-acetate; peak 10: alisol B 23-acetate; and peak 12: alisol O isomer.

We performed preliminary identification of compounds 3, 4, 5, 7, 10, and 12 using existing standards in the laboratory. It was confirmed that peaks 3, 4, 5, 7, and 10 were alisol F, alisol C 23-acetate, alisol A, alisol A 24-acetate, and alisol B 23-acetate, respectively. Compound 12 was identified based on relative polarity size, precise molecular mass, secondary debris, and references to previous studies that the mass error of the molecular ions of all compounds was within ±3 ppm (Table 1). The six components obtained via the analysis were all tetracyclic triterpenoids, most of which were pro-terpene types. Triterpenoids were rearranged by C-23–C-24 cleavage, and a series of key backbone ions such as $[M + H\text{-}H_2O]^+$, $[M + H\text{-}HA_C]^+$, $[M + H\text{-}2H\text{-}2H\text{-}HA_C]^+$, and so forth were produced by a series of lost H_2O or HA_C (60 Da). The lysis law of each compound was analyzed as follows.

Table s1. Identification of the chemical constituents of the SAR extract using UPLC–QTOF–

MS/MS in positive ion mode

No.	Rt (min)	$[M + H]^+$/$[M + Na]^+$ theoretical	Error (ppm)	$[M + H]^+$ /$[M + Na]^+$ ion	Fragmentions	Molecular formula	Identity
3	22.30	511.3399	1.6	$[M + Na]^+$	511.3401, 489.3623, 471.3488, 453.3367, 435.3242, 381.2791, and 339.2698	$C_{30}H_{48}O_5$	alisol F
4	22.73	529.3538	1.7	$[M + H]^+$	529.3536, 511.3423, 469.3324, 451.3217, and 433.3113	$C_{32}H_{48}O_6$	alisol C 23-acetate
5	26.17	513.3556	0	$[M + Na]^+$	513.3562, 473.3638, 455.3533, 383.2952, 365.2842, and 339.2690	$C_{30}H_{50}O_5$	alisol A
7	28.89	555.3662	–2.2	$[M + Na]^+$	555.3655, 515.3717, 497.3627, 383.2931, 365.2846, and 339.2690	$C_{32}H_{52}O_6$	alisol A 24-acetate
10	34.15	515.3736	0.8	$[M + H]^+$	515.3743, 497.3633, 437.3422, 419.3314, 383.2957, 365.2847, and 339.2688	$C_{32}H_{50}O_5$	alisol B 23-acetate
12	36.98	513.358	–0.4	$[M + H]^+$	535.3376, 495.3439, 453.3355, 435.3254, and 381.2784	$C_{32}H_{48}O_5$	alisol O isomer

Compound 3 had a retention time of 22.30 min and a molecular formula of $C_{30}H_{48}O_5$. It showed fragment ions at *m/z* 511.3407 $[M + Na]^+$, 471.3488 $[M + H\text{-}H_2O]^+$, 453.3367 $[M + H\text{-}2H_2O]^+$, 435.3242 $[M + H\text{-}3H_2O]^+$, 381.2791 $[M + H\text{-}C_4H_{12}O_3]^+$, and 339.2698 $[M + H\text{-}C_6H_{14}O_4]^+$ in the positive ion mode. According to the fragmentation information combined with the reference, it was identified as alisol F. The fragmentation process is shown in Figure 2.

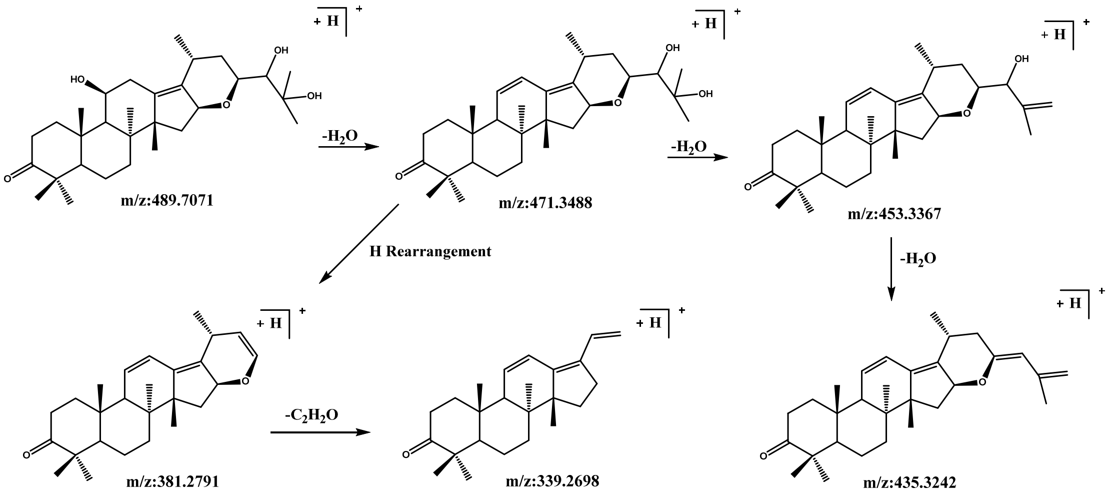

Figure s2. Fragmentation process of alisol F in the positive ion mode.

In electrospray ionization (ESI) positive ion mode, compound 4 exhibited a retention time of 22.73 min and a molecular formula of $C_{32}H_{48}O_6$. It produced major fragment ions at *m/z* 529.3529 $[M + H]^+$, 511.3423 $[M + H\text{-}H_2O]^+$,469.3324 $[M + H\text{-}HAc]^+$, 451.3217 $[M + H\text{-}H_2O\text{-}HAc]^+$, and 433.3113 $[M + H\text{-}2H_2O\text{-}HAc]^+$. Based on the retention time, fragment ions, and comparison with known compounds, it was confirmed that the compound was alisol C 23-acetate. The fragmentation process is shown in Figure 3.

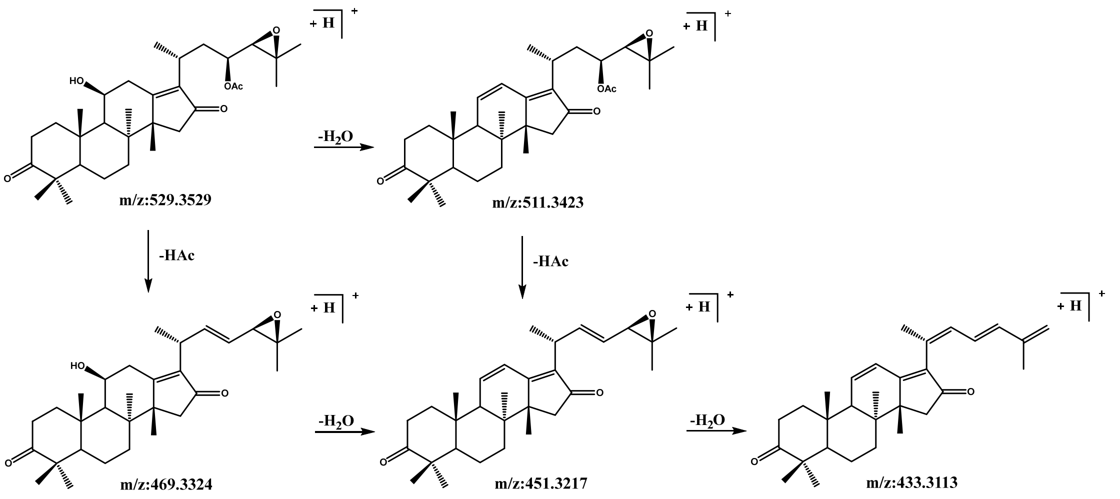

Figure s3. Fragmentation process of alisol C 23-acetate in the positive ion mode.

Compound 5, with a retention time of 26.17 min and a molecular formula of $C_{30}H_{50}O_5$, exhibited fragment ions at *m/z* 513.3556 $[M + Na]^+$, 473.3638 $[M + H\text{-}H_2O]^+$, 455.3533 $[M + H\text{-}2H_2O]^+$, 383.2952 $[M + H\text{-}H_2O\text{-}C_4H_{10}O_2]^+$, 365.2842 $[M + H\text{-}C_4H_{14}O_4]^+$, and 339.2690 $[M + H\text{-}C_6H_{16}O_4]^+$ in the positive ion mode. Based on the fragmentation information and previous studies, the compound was identified as alisol A. The specific cracking process for alisol A is shown in Figure 4.

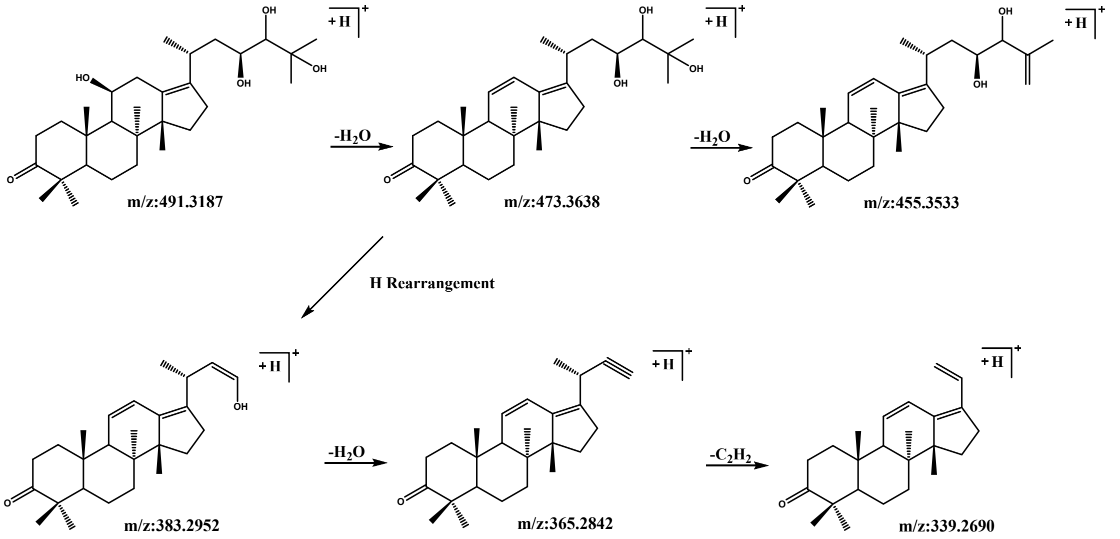

Figure s4. Fragmentation process of alisol A in the positive ion mode.

Compound 7 exhibited a retention time of 28.89 min and a formula of $C_{32}H_{52}O_6$. Several main fragment ions at *m/z* 555.3650 $[M + Na]^+$, 515.3717 $[M + H\text{-}H_2O]^+$, 497.3627 $[M + H\text{-}2H_2O]^+$, 383.2931 $[M + H\text{-}H_2O\text{-}C_6H_{12}O_3]^+$, 365.2846 $[M + H\text{-}C_6H_{16}O_5]^+$, and 339.2690 $[M + H\text{-}C_8H_{18}O_5]^+$ were observed in the positive ion mode. Based on the fragmentation information and previous studies, the compound was identified as alisol A 24-acetate. The specific cracking process for alisol A 24-acetate is shown in Figure 5.

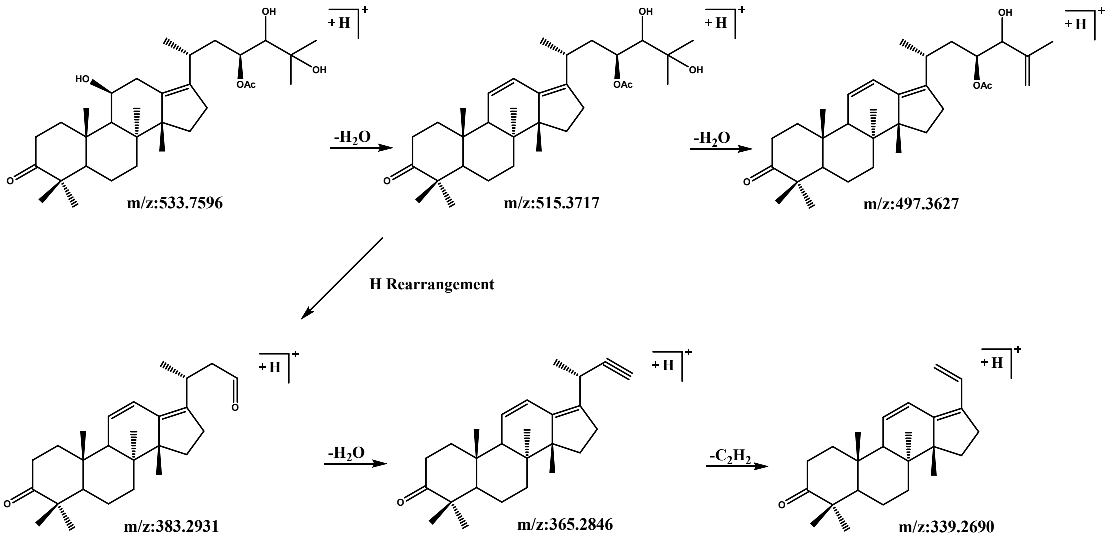

Figure s5. Fragmentation process of alisol A 24-acetate in the positive ion mode.

Compound 10 had a retention time of 34.15 min and a molecular formula of $C_{32}H_{50}O_5$. It showed fragment ions at *m/z* 515.3740 $[M + H]^+$, 497.3633 $[M + H\text{-}H_2O]^+$, 437.3422 $[M + H\text{-}H_2O\text{-}HAc]^+$, 419.3314 $[M + H\text{-}2H_2O\text{-}HAc]^+$, 383.2957 $[M + H\text{-}H_2O\text{-}C_6H_{10}O_2]^+$, 365.2847 $[M + H\text{-}C_6H_{14}O_4]^+$, and 339.2688 $[M + H\text{-}C_8H_{16}O_4]^+$ in the positive ion mode. According to the fragmentation information and previous studies, the compound was identified as alisol B 23-acetate. The fragmentation process is shown in Figure 6.

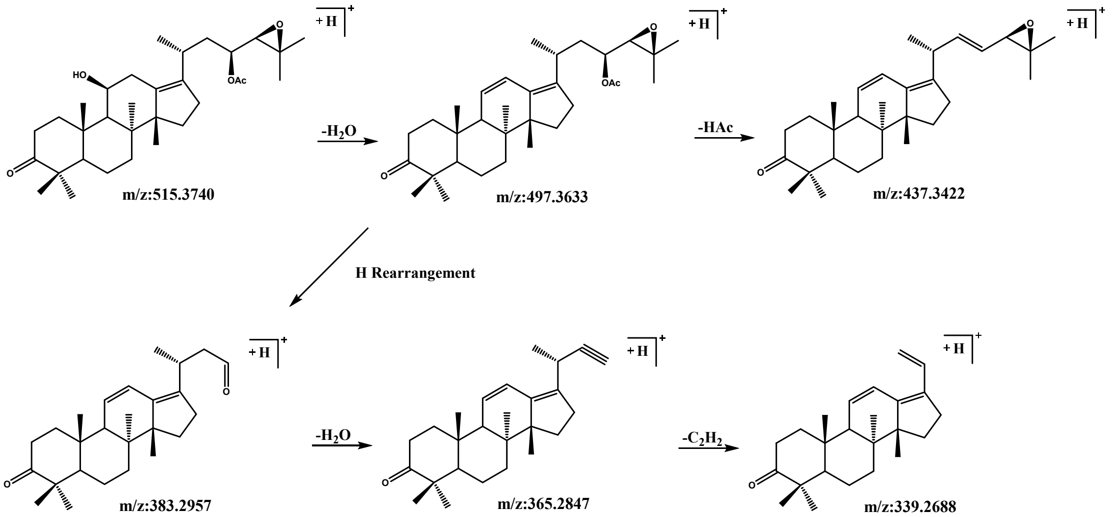

Figure s6. Fragmentation process of alisol B 23-acetate in the positive ion mode.

Compound 12, with a retention time of 36.98 min and a molecular formula of $C_{32}H_{48}O_5$, exhibited fragment ions at *m/z* 513.3578 $[M + H]^+$, 495.3439 $[M + H\text{-}H_2O]^+$, 453.3353 $[M + H\text{-}HAc]^+$, 435.3254 $[M + H\text{-}H_2O\text{-}HAc]^+$, and 381.2784 $[M + H\text{-}C_6H_{12}O_3]^+$ in the positive ion mode. Based on the fragmentation information and previous studies, the compound was identified as an alisol O isomer. The specific cracking process for the alisol O isomer is shown in Figure 7.

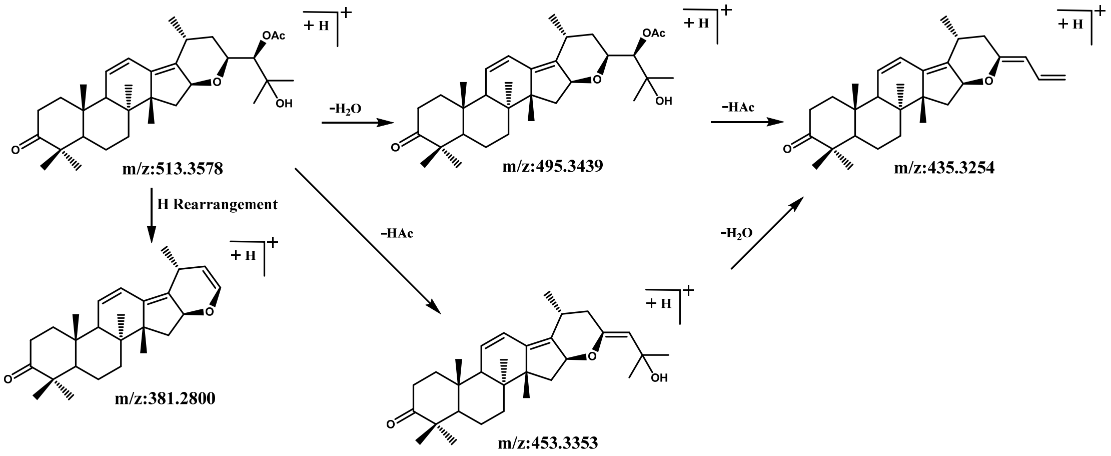

Figure s7. Fragmentation process of alisol O isomer in the positive ion mode.

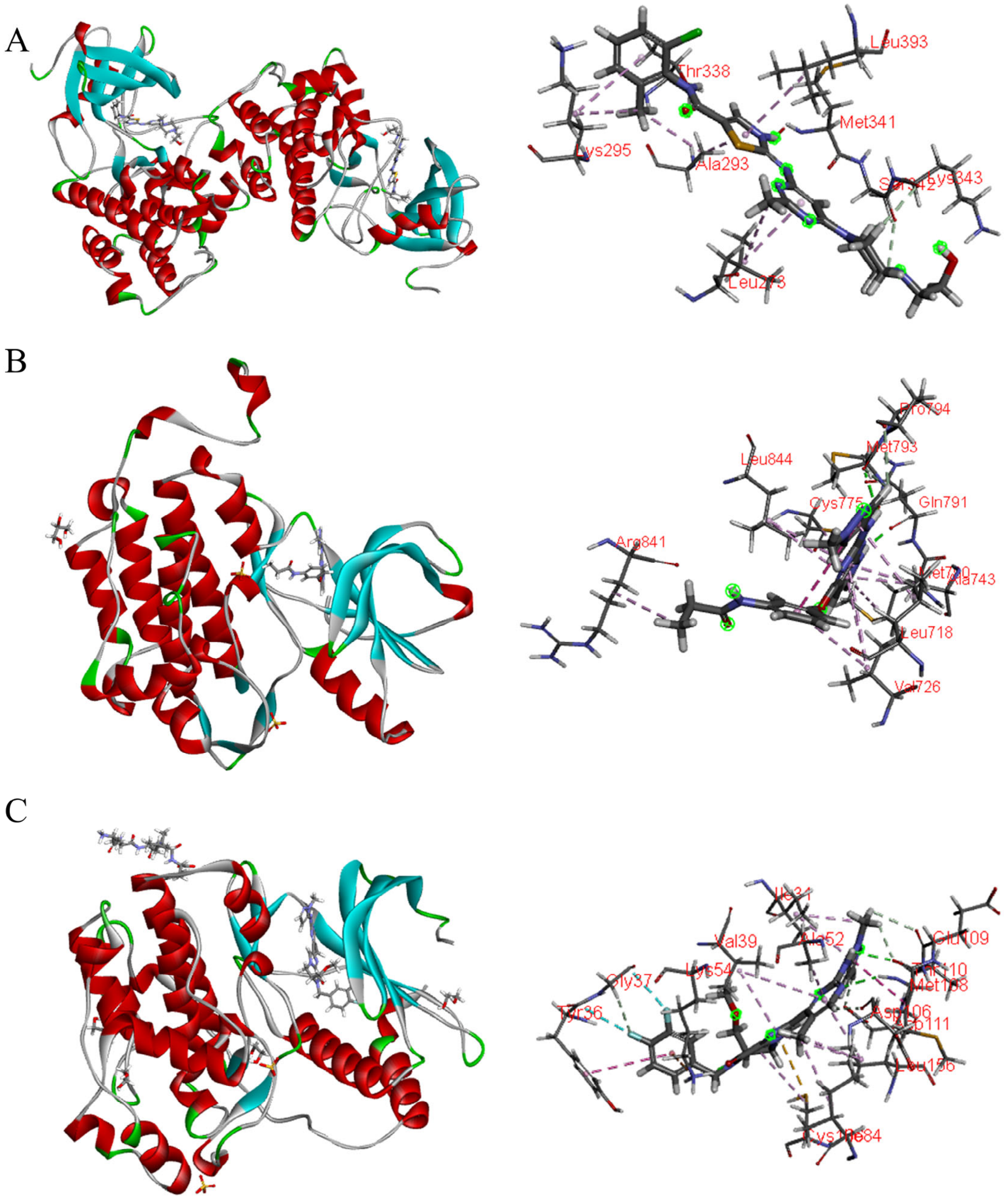

Figure s8. 3D Diagram of protein and original ligand interaction (A: 3QLG; B: 5HG8; C: 6SLG)

Table s2 The HPLC elution gradient

t/min	Acetonitrile (A) /%	Water (B) /%	Methanol (C) /%
0	35	60	5
2	35	60	5
5	50	46	4
8	50	46	4
40	65	32	3
75	65	32	3
85	90	9	1
95	90	9	1
96	35	60	5
105	35	60	5
